# *Cryptococcus gattii* infection complicated by immune reconstitution syndrome in a patient with common variable immunodeficiency

**DOI:** 10.1590/0037-8682-0443-2022

**Published:** 2022-12-16

**Authors:** Luize Meloti Fiorio, Claudia Biasutti, Marcos Rosa-Júnior

**Affiliations:** 1 Universidade Federal do Espírito Santo, Centro de Ciências da Saúde, Vitória, ES, Brasil.; 2 Hospital Universitário Cassiano Antônio Moraes, Setor de Infectologia, Vitória, ES, Brasil.; 3 Hospital Universitário Cassiano Antônio Moraes, Setor de Neurorradiologia, Vitória, ES, Brasil.

**FIGURE 1:**
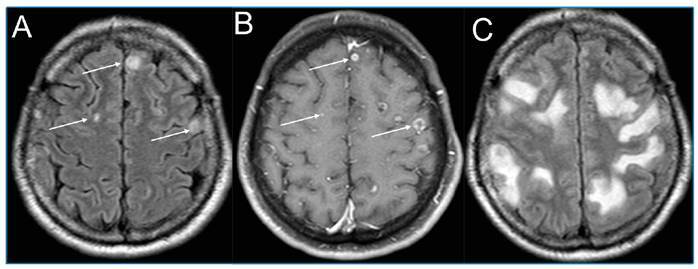
**(A)** MRI shows small peripheral lesions on FLAIR. **(B)** MRI shows small peripheral lesions with annular contrast enhancement on T1WI post-contrast. **(C)** The new MRI shows increased expansive mass effect and vasogenic edema on FLAIR. FLAIR: fluid attenuated inversion recovery. T1WI: T1-weighted image.

Cryptococcosis is a fungal disease caused by *Cryptococcus sp* ^1^. *C. neoformans* mainly affects immunocompromised patients, whereas *C. gattii* affects immunocompetent patients^1^. We present the case of a 51-year-old man with common variable immunodeficiency, a hypogammaglobulinemia with reduced serum concentrations of IgG, IgA, or IgM, which led to susceptibility to infections. He was diagnosed with C. gattii infection in the central nervous system (CNS) presenting with immune reconstitution syndrome (IRIS), headache, and recurrent episodes of paresthesia and seizures. Brain magnetic resonance imaging (MRI) revealed multiple cystic lesions with peripheral gadolinium enhancement (Figure 1A and 1B). During treatment, he developed IRIS (Figure 1C), a paradoxical worsening of radiologic and clinical features, which is a relevant complication of cryptococcosis treatment commonly described in HIV-infected patients. Therefore, dexamethasone therapy (16 mg/dL) was initiated to treat the IRIS-like presentation. A repeat MRI after 20 days showed improvements.

The radiologic and clinical deterioration following 2 weeks of treatment, despite mycological evidence of effective antifungal therapy in the CSF, had a paradoxical IRIS-like response. IRIS-like syndrome was first described in patients with HIV after initiating antiretroviral therapy and was also reported in *C. gattii* infections^2^ ^,^ ^3^. It results from an initial Th2 cytokine response that transitions to an excessive Th1 response during therapy^3^. Corticosteroids are not part of the treatment for cryptococcosis; however, in this case, they may minimize CNS inflammation and reduce symptoms.

Initially, the patient recovered clinically and radiologically following amphotericin B, fluconazole, and dexamethasone therapy with no neurological deficits; however, he later died of complications during hospitalization.
